# Comparative Transcriptomic Analysis of Regenerated Skins Provides Insights into Cutaneous Air-Breathing Formation in Fish

**DOI:** 10.3390/biology10121294

**Published:** 2021-12-08

**Authors:** Songqian Huang, Bing Sun, Longfei Huang, Lijuan Yang, Chuanshu Liu, Jinli Zhu, Jian Gao, Xiaojuan Cao

**Affiliations:** 1 Key Lab of Freshwater Animal Breeding, Ministry of Agriculture, College of Fisheries, Huazhong Agricultural University, Wuhan 430070, China; huangsongqian0115@gmail.com (S.H.); sunbing931014@163.com (B.S.); 15207173315@163.com (L.H.); atyanglijuan@163.com (L.Y.); liuchuanshu2021@163.com (C.L.); 2 Department of Aquatic Bioscience, Graduate School of Agricultural and Life Sciences, The University of Tokyo, Bunkyo-ku, Tokyo 113-8657, Japan; 3 Engineering Research Center of Green Development for Conventional Aquatic Biological Industry in the Yangtze River Economic Belt, Ministry of Education/Hubei Provincial Engineering Laboratory for Pond Aquaculture, College of Fisheries, Huazhong Agricultural University, Wuhan 430070, China; 4 National Demonstration Center for Experimental Aquaculture Education, Huazhong Agricultural University, Wuhan 430070, China; zhujinli1898@163.com

**Keywords:** cutaneous air-breathing, skin regeneration, comparative transcriptome, *Misgurnus anguillicaudatus*, *Pelteobagrus fulvidraco*

## Abstract

**Simple Summary:**

Cutaneous air-breathing is one of the air-breathing patterns in bimodal respiration fishes, while little is known about its underlying formation mechanisms. The skin regeneration of loach (*Misgurnus anguillicaudatus*, a cutaneous air-breathing fish) and yellow catfish (*Pelteobagrus fulvidraco*, a water-breathing fish) were first investigated through morphological and histological observations. Then, the original skins (OS: MOS, POS) and regenerated skins (RS: MRS, PRS) when their capillaries were the most abundant during healing, of the two fish species were collected for high-throughput RNA-seq. A total of 56,054 unigenes and 53,731 unigenes were assembled in loach and yellow catfish, respectively. A total of 640 (460 up- and 180 down-regulated) and 4446 (2340 up- and 2106 down-regulated) differentially expressed genes (DEGs) were respectively observed in RS/OS of loach and yellow catfish. Subsequently, the two DEG datasets were clustered in GO, KOG, and KEGG databases, and further analyzed by comparison and screening. Consequently, tens of genes and thirteen key pathways were targeted, indicating that these genes and pathways had strong ties to cutaneous skin air-breathing in loach. This study provides new insights into the formation mechanism of cutaneous air-breathing and also offers a substantial contribution to the gene expression profiles of skin regeneration in fish.

**Abstract:**

Cutaneous air-breathing is one of the air-breathing patterns in bimodal respiration fishes, while little is known about its underlying formation mechanisms. Here, we first investigated the skin regeneration of loach (*Misgurnus anguillicaudatus*, a cutaneous air-breathing fish) and yellow catfish (*Pelteobagrus fulvidraco*, a water-breathing fish) through morphological and histological observations. Then, the original skins (OS: MOS, POS) and regenerated skins (RS: MRS, PRS) when their capillaries were the most abundant (the structural foundation of air-breathing in fish) during healing, of the two fish species were collected for high-throughput RNA-seq. A total of 56,054 unigenes and 53,731 unigenes were assembled in loach and yellow catfish, respectively. A total of 640 (460 up- and 180 down-regulated) and 4446 (2340 up- and 2106 down-regulated) differentially expressed genes (DEGs) were respectively observed in RS/OS of loach and yellow catfish. Subsequently, the two DEG datasets were clustered in GO, KOG and KEGG databases, and further analyzed by comparison and screening. Consequently, tens of genes and thirteen key pathways were targeted, indicating that these genes and pathways had strong ties to cutaneous skin air-breathing in loach. This study provides new insights into the formation mechanism of cutaneous air-breathing and also offers a substantial contribution to the gene expression profiles of skin regeneration in fish.

## 1. Introduction

It is well acknowledged that gill respiration is not the only way for fish to obtain oxygen. So far, about 450 fish species have been reported to perform assistant air-breathing [1]. Air-breathing fish take oxygen from air by using their auxiliary air-breathing organs (ABOs) rich in blood vessels, such as skin, buccal and pharyngeal cavity, swim-bladder, tail fin, as well as digestive tract [2,3]. Research on fish of bimodal respiration mainly focuses on morphology and histology of ABOs, respiratory physiology, and their aquaculture applications [4,5,6]. It has been found that high capillary vascularization is the histological basis for fish ABOs.

Molecular mechanisms of air-breathing formation in fish are still poorly understood and little literature has been published. A transcriptome analysis of *Channa argus* involved gill and the suprabranchial chamber (an ABO) was performed, and then screened out a batch of genes probably related to the formation of air-breathing, such as vascular endothelial growth factor (*vegf*) and fibroblast growth factor receptor (*fgfr*) [7]. The whole genome sequencing, assembly, and annotation of *C. argus* were carried out, expecting to unearth the key genes involved in air-breathing regulation [8]. A comparative transcriptome study of walking catfish *Clarias batrachus* showed that the hemoglobin gene and angiogenesis-related genes were obviously up-regulated in its ABO relative to the gill [9]. In total, 15 key pathways and 25 key genes were identified by a developmental transcriptome analysis, presenting relationship to the formation of intestinal air-breathing in loach (*Misgurnus anguillicaudatus*) in our previous research [10]. Moreover, after inhibiting the intestinal air-breathing in loach, we mined some potential genes and miRNAs important for maintaining the air-breathing [11,12]. At present, large number of genes and pathways have been proven involved in gas exchange process and vascularization in vertebrate [13,14,15], but it is rarely reported to attach these genes and pathways to fish air-breathing formation.

Loach *M. anguillicaudatus*, a small freshwater teleost fish that lives in rice fields and streams, and is widespread in Eurasia [16], can exchange gas both in water via the gill, and in air via intestine and skin [17,18]. The skin is considered as an auxiliary respiratory organ in loach, while the attention paid to it primarily lies in the aspects of morphology and histology [19,20,21]. To satisfy the demand of cutaneous respiration, the main modifications targeted on the skin of loach are as follows: high capillary epithelialization, degenerated scales, and well differentiated cutaneous gland cells. In addition, the middle layer of loach skin epidermis is mainly composed of multi-laminar swollen cells, which serve as a barrier to effectively reduce the moisture loss [22] and avoid dry skin when exposing to air for a long time. Yellow catfish (*Pelteobagrus fulvidraco*), a small freshwater teleost fish without air-breathing function, shares common niche and diet with the loach [23,24]. In addition, skins of these two fishes are rich in goblet cells, capable of secreting a large amount of mucus, especially under stress, although the skin surface of yellow catfish is lacking tiny scales like the loach [21,23].

In the present study, skin regeneration observations of loach and yellow catfish were first performed by virtue of skin incision, morphological, and histological methods. Next, regenerated skins when their highest vascularization occurred and original skins of the two fishes were collected for RNA-seq and comparative analysis, aiming to investigate the molecular mechanism of cutaneous air-breathing formation in the loach. This study not only provides a novel understanding of the mechanisms of cutaneous air-breathing formation, but also offers a substantial contribution to the gene expression profiles of skin regeneration in fish.

## 2. Materials and Methods

### 2.1. Fish

All experimental diploid loach *M. anguillicaudatus* (the average weight and body length were 13.40 ± 0.51 g and 11.77 ± 1.81 cm, respectively) and yellow catfish *P. fulvidraco* (the average weight and body length were 86.75 ± 12.85 g and 17.40 ± 1.17 cm, respectively) were obtained from the Aquaculture Base of College of Fisheries, Huazhong Agricultural University in Wuhan City, Hubei Province, China. The experimental fish were cultured in recirculating water with temperature of 24–26 °C and dissolved oxygen of 6.5 ± 0.7 mg·L^−1^ and fed three times a day.

### 2.2. Cutaneous Air-Breathing Confirmation in Loach

It is thought that loach can breathe air via its skin, but it has not been confirmed by experiments. Since loach could inhale air through its mouth to posterior intestine conducting accessory respiration, mouth suture method was carried out in loach to restrain intestinal air-breathing, and then the cutaneous air-breathing of the loach was confirmed (Figure S1). Firstly, the mouths of loaches were stitched with surgical sutures (Figure S1a). Then, we placed the loaches in water in a sealed plastic bag (Figure S1b, 2/3 volume was water and 1/3 was nitrogen). After asphyxia by hypoxia, we removed half of the loaches and exposed them to air (Figure S1c,d, the air exposure group), which had neither waterbreathing, nor intestinal air-breathing at this time. The other half of loaches remained in the plastic bag without dissolved oxygen in the water (the control group). The survival rates of loaches were evaluated at 15 min after air exposure and non-air exposure.

### 2.3. Cutaneous Incision and Morphological Observations of Regenerated Skins

**Figure 1:**
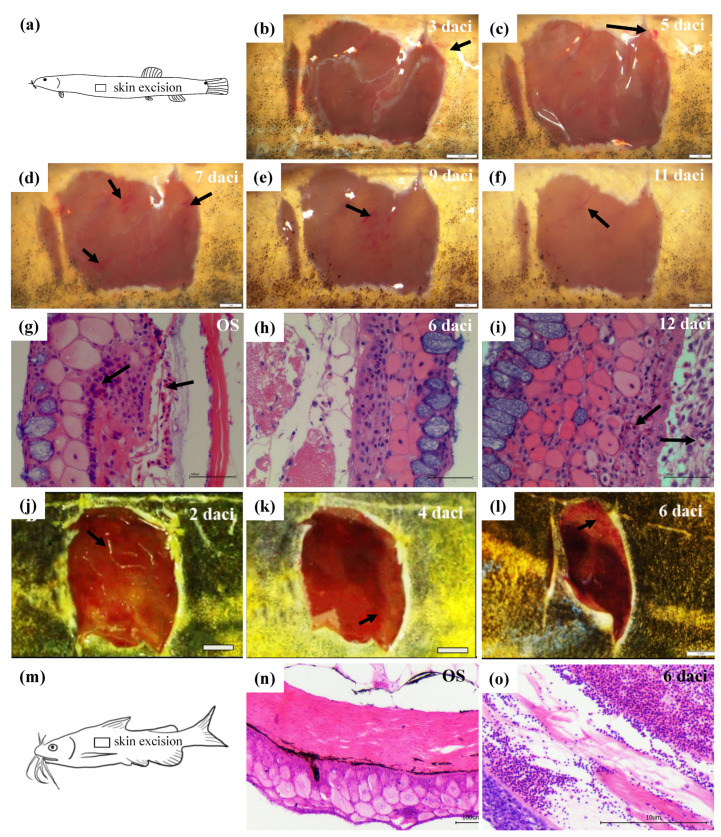
Morphological and histological observations of regenerated skins of loach and yellow catfish. (**a**) Sketch map of cutaneous incision for morphological observations of regenerated skins. (**b**–**f**) Morphologies of regenerated skins of the 3rd, 5th, 7th, 9th, and 11th day after cutaneous incision (daci) of loach. Scale bars indicate 1 mm. (**g**–**i**) Histological structures of original skin (OS) and regenerated skins of the 6th and 12th daci of loach. Scale bars indicate 10 μM. Black arrows show blood vessels. (**j**–**l**) Morphologies of regenerated skins of the 2nd, 4th, and 6th daci of yellow catfish. Scale bars indicate 1 mm. (**m**) Sketch map of cutaneous incision for morphological observations of regenerated skins. (**n**,**o**) Histological structures of original skin (OS) and 6th daci regenerated skin in yellow catfish.

After anesthetized with 100 mg·L^−1^ tricaine methanesulfonate (MS-222) [11], the skin tissues of 1 cm^2^ size beneath the dorsal fin of ten loaches were dissected away by virtue of sharp and sterile blades (Figure 1a). After a few minutes of recovery in fresh aerated water, the loaches were held temporarily in an anti-infection tank by adding with 1 mg·L^−1^ malachite green dye (Sigma-Aldrich, St. Louis, MO, USA), next day the survival fish were returned to the rearing system [25]. Then, the process of loach skin regeneration and development was observed and photographed under the stereomicroscope (Nikon SMZ 1500, Tokyo, Japan) every day, till the wound was completely healed. The identical operation above was entirely applied in yellow catfish simultaneously.

### 2.4. Cutaneous Incision and Histological Observations of Regenerated Skins

According to the morphological observation results of regenerated skins in loach and yellow catfish, the original skin and the regenerated skins of 3rd, 6th, 9th, and 12th day after cutaneous incision (daci) of loach (*n* = 5), and the original skin and the regenerated skins of 2nd, 4th, and 6th daci of yellow catfish (*n* = 5) were dissected out for histological observations. The skin tissues were fixed in the Bouin’s fixative for 24 h and then stored in 70% ethanol. After dehydration in a graded series of ethanol and transparency by xylene, the skin samples were embedded in paraffin and sectioned in serial transverse sections (5 μm thick), by using a Leica RM 2135 rotary microtome (Leica Ltd., Wetzlar, Germany). Dewaxed serial sections were stained with Delafield’s haematoxylin for 24 h, then all samples were dehydrated in graded ethanol and embedded in paraffin wax. Cross sections of 4 μm thickness were stained with hematoxylin and eosin (H&E) to show general histological observations of the regenerated skins of loach and yellow catfish [24].

### 2.5. RNA Isolation and cDNA Library Constructions

According to the morphological and histological observations, the optimal sampling time points of the regenerated skins (when cutaneous capillaries were the most abundant during healing, which was the structural foundation of air-breathing in fish) for transcriptome analysis were determined. For loach, the 10th daci regenerated skin (MRS) and the original skin (MOS) on the other side symmetrical along the axis of the body were simultaneously taken, with three sets of parallels. Meanwhile, for yellow catfish, the 5th daci regenerated skin (PRS) and the original skin (POS) on the other side symmetrical along the axis of the body were simultaneously taken, with three sets of parallels.

Total RNA was isolated from the skin tissues according to the manufacturer’s protocol. The extracted RNA samples of high qualities were used for the cDNA synthesis. Poly (A) mRNA was isolated by using oligo-dT beads (Qiagen, Redwood City, CA, USA). All mRNA was sheared into short fragments (200 nt) by adding fragmentation buffer. First-strand cDNA was generated using random hexamer-primed reverse transcription, followed by the synthesis of the second-strand cDNA using RNase H and DNA polymerase I. The cDNA fragments were purified by using a QIAquick PCR extraction kit. These purified fragments were then washed with EB buffer for end reparation poly (A) addition and ligated to sequencing adapters. Following agarose gel electrophoresis and extraction of cDNA from gels, the cDNA fragments (200 ± 5 bp) were purified and enriched by PCR to construct the cDNA library (PE100). Finally, six cDNA libraries (MOS1, MOS2, MOS3, MRS1, MRS2, and MRS3) were constructed in loach; so did yellow catfish (POS1, POS2, POS3, PRS1, PRS2, and PRS3).

### 2.6. Sequencing, De Novo Assembly and Functional Annotation

The cDNA libraries were sequenced on the Illumina sequencing platform (Illumina HiSeq™ 2500) using the paired-end technology in a single run. The Illumina GA processing pipeline was used to analyze the image and for base calling. The high-quality sequences were required for de novo assembly analysis. Before assembly, raw sequencing reads were trimmed by removing adapter sequences and low-quality nucleotides using FastQC (version 0.11.5) and Cutadapt (-a, Version 1.9.1). Then, all clean reads of the libraries of same species were jointly assembled into contigs performed by Trinity software (version 2.4.0) [26]. After assembly, contigs longer than 200 bases were used for subsequent analysis. The contigs were connected to access the sequence that could not be extended on either end, and the sequence of the unigene was then produced. Next, the unigenes were further spliced and assembled to obtain maximum length non redundant unigenes using TGICL clustering software (-F, version 2.1). Finally, unigenes were aligned with the NCBI Nr [27], Swiss-Prot [28], GO [29], KOG [30], and KEGG [31] database using Blastx procedure in blast (version 2.2.26) with an E-value < 10^−5^. Blast2GO (version 5.2.5) was used to obtain GO annotation of the unigenes based on Blastx hits against the NCBI Nr database. Blastn was used to align the unigenes to the NCBI Nr nucleotide database, retrieving proteins with the highest sequence similarity with the given unigenes, along with their protein functional annotations.

### 2.7. Differentially Expressed Gene (DEG) Analysis and Enrichment Analysis

The mapped fragments were normalized for RNA length according to fragment per kilobase of exon model per million mapped reads (FPKM) for each gene [32], which facilitated the comparison of transcript levels between samples. DEGs between MOS and MRS, and between POS and PRS were identified by the DEG-seq package (R software, version 3.4.0) using the MA-plot-based method with Random Sampling model (MARS) method [33]. DEGs were selected by using the following filter criteria: false discovery rate (FDR) <0.05 and the absolute value of log_2_FC (fold change) >1, meaning that the expression difference for each DEG in different libraries should be at least two-fold. Furthermore, the enrichment analysis of DEGs was conducted with GO database, and the gene number of each GO term was calculated. The main pathways of biochemical and signal transduction significantly associated with DEGs were determined via a KEGG pathway analysis. Finally, the DEGs, which associated with air-breathing and regenerative function, were analyzed in detail.

### 2.8. Mining of DEGs Related to Cutaneous Air-Breathing

After the acquisition of two DEGs settings from loach and yellow catfish, the genes of quite significant differences between the two data settings were specially selected as candidate genes and listed in an Excel sheet. Subsequently, subtractive analysis was performed: the same genes happened to loach and yellow catfish were removed from the candidate genes. The rest DEGs of loach were considered to have tight attachment to cutaneous air-breathing, rather than skin regeneration. Furthermore, tens of potential cutaneous air-breathing formation related genes were selected from the DEGs of loach and validated in different loach skin tissues (including OS and regenerated skins of 3rd, 6th, 9th, and 12th daci) by quantitative PCR (qPCR). The primers were designed based on the assembly gene sequences (Table S1).

### 2.9. Validation of Transcriptome Data by qPCR

To examine the reliability of the transcriptome results, six DEGs (fibronectin1 (*fn1*), integrinα5 (*itga5*), lysyl oxidase-like 2a (*loxl2a*), *loxl2b*, lectin galactoside-binding-like 1 (*lgals1*), and pleckstrin homology H1 (*plekhh1*)) and six DEGs (*fn1*, *itga5*, *loxl2a*, chemokine receptor 4 (*cxcr4*), *vegfaa*, and transforming growth factor-beta receptor 2 (*tgfbr2*)) in loach and yellow catfish were, respectively, selected to perform qPCR. Total RNAs isolated from the original and regenerated skins were reverse transcribed to first-strand cDNA using reverse transcriptase (Invitrogen, Waltham, MA, USA). The qPCR was carried out on an iQ5 system (Bio-Rad, Hercules, CA, USA) using SYBR Premix Ex Taq (TaKaRa, Dalian, China), according to the manufacturer’s instructions. The primers for these genes were designed manually (Table S1). The reaction mixture (10 μL) comprised 2.5 μL cDNA (1:4 dilution), 5 μL SYBR Premix Ex TaqTM II (TaKaRa), 0.5 μL specific forward primer (10 μM), 0.5 μL universal primer (10 μM), and 1.5 μL water. The reactions were performed in an MJ Opticon™-2 machine (Bio-Rad, Hercules, CA, USA). The relative expression levels were normalized to the endogenous control gene *β-actin*, and calculated by the 2^−ΔΔCt^ method [34].

### 2.10. Statistical Analysis

All qPCR data were presented as the means of three individual experiments ± standard deviations. Statistical analysis was conducted by *t* test using SPSS 19.0 (Michigan Avenue, Chicago, IL, USA). Probability values of *p* < 0.05 was considered statistically significant.

## 3. Results

### 3.1. Morphological and Histological Observations of Skin Regeneration

In the experiment of loach cutaneous air-breathing confirmation, after hypoxia treatment, the air-exposed loaches with intestinal air-breathing inhibited had a significant higher survival rate than the control group (Figure S1e), which indicated that there was cutaneous air-breathing in the loach. The epidermal skin fragments were dissected away by surgical operation (Figure 1a). The vascularization of the cutting area edges was obvious on the 3rd daci in loach (Figure 1b). Blood capillaries spread to the whole regeneration area of skin on the 5th daci (Figure 1c). On the 7th daci, the vascularization of the regenerated cutaneous edges reverted to the normal level (Figure 1d). Then, till the 9th daci, the vascularized area of the regenerated skin had been transferred to the central part (Figure 1e). Finally, the vascularization of the regenerated skin on the 11th daci was totally completed (Figure 1f).

Furthermore, the histological observations of skin regeneration were performed in loach. Generally, the capillaries are mostly detected in the loose layer of dermis, a few of which stick to the germinal layer or even extend to the epidermis in the original skin (Figure 1g). During the process of cutaneous regeneration, the epidermal layer of the skin appeared completely on 3rd daci, with multi-laminar mucous cells and swollen cells, but without dermis differentiation. On the 6th daci, the muscularis began to separate from the epidermis, with emerge of the dermal loose layer (Figure 1h). The muscular layer and the epidermis layer were remarkably separated on the 9th daci, with rather abundant capillaries permeating in it. Finally, on the 12th daci, the germinal layer showed an obvious structure of single-layered cubic cells or columnar cells, and the capillaries within the loose layer were more than those on the 9th daci, reaching the maximum (Figure 1i). Morphologically and histologically, yellow catfish went through similar skin regeneration process as loach (Figure 1j–o). On the 6th daci, the skin regeneration in yellow catfish was basically completed (Figure 1l). No obvious capillaries distributed in the original and regenerated skins of yellow catfish were observed (Figure 1n,o).

### 3.2. De Novo Assembly and Functional Annotation

A total of twelve libraries of original and regenerated skins of loach and yellow catfish were performed by RNA-seq. After cleaning and quality checks, the RNA-seq produced 266.13 million clean reads with a total of 36.07 Gb for loach, and 291.48 million clean reads with a total of 44.83 Gb for yellow catfish (Table 1). A total of 56,056 unigenes with a mean length of 991 bp and 53,731 unigenes with a mean length of 1080 bp were, respectively, identified in loach and yellow catfish.

To annotate the sequences of loach and yellow catfish, searches were conducted against five public databases (Nr, SwissProt, KEGG, KOG, and GO) (Figure S2). In total, 32,684 (58.31%) unigenes of loach and 27,169 (52.43%) unigenes of yellow catfish were simultaneously annotated in more than one public databases. All unigenes from OSs and RSs of loach and yellow catfish were used for function enrichment and classifications analysis. Together, 42,250 (75.37%) unigenes of loach and 39,676 (73.84%) unigenes of yellow catfish were both annotated in KOG and grouped into 25 KOG classifications which presented almost identical distribution regularity between loach and yellow catfish, especially the top five items. The largest cluster was the signal transduction mechanisms (21.62% unigenes from loach, and 22.20% unigenes from yellow catfish), and then followed by general function prediction only (18.08% unigenes from loach, and 17.17% unigenes from yellow catfish), posttranslational modification—protein turnover—chaperones (8.92% unigenes from loach, and 8.72% unigenes from yellow catfish), transcription (5.87% unigenes from loach, and 6.33% unigenes from yellow catfish), and intracellular trafficking—secretion—vesicular transport (5.13% unigenes from loach, and 5.66% unigenes from yellow catfish).

GO analysis of all unigenes showed that a total of 17,008 (30.34%—loach) and 15,611 (29.05%—yellow catfish) unigenes were annotated. In detail, cellular process (5891 unigenes from loach and 7946 unigenes from yellow catfish) and single-organism process (5416 unigenes from loach and 7105 unigenes from yellow catfish) represented the majority category of biology process. Cell and cell part represented the majority category of cellular component. Moreover, binding and catalytic activity (4229 unigenes from loach and 5654 unigenes from yellow catfish) represented a high percentage of the molecular function category.

For further identification of the biological pathways in loach and yellow catfish, we mapped all the unigenes to the reference of typical pathways in the KEGG database. 15,929 unigenes (28.42%) of loach and 14,372 unigenes (26.75%) of yellow catfish were matched to 230 and 223 different KEGG pathways, respectively. Among these unigenes, top four enrichment KEGG pathways in loach and yellow catfish were identical including focal adhesion (ko04510, 489 unigenes, top1 in loach; 347 unigenes, top4 in yellow catfish), endocytosis (ko04144, 480 unigenes, top2 in loach; 453 unigenes, top1 in yellow catfish), MAPK signaling pathway (ko04010, 424 unigenes, top3 in loach; 402 unigenes, top2 in yellow catfish) and regulation of actin cytoskeleton (ko04810, 404 unigenes, top4 in loach; 376 unigenes, top3 in yellow catfish).

### 3.3. DEG Analysis and Enrichment Analysis

**Figure 2:**
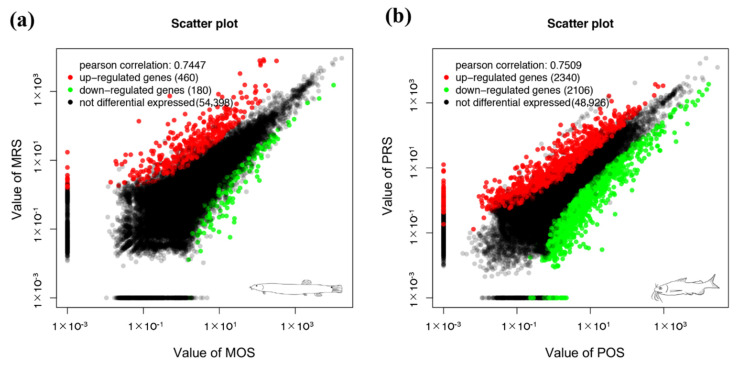
Scatter plot map for genes in comparisons of RS/OS pertain to loach (**a**) and yellow catfish (**b**). Each plot represents an individual gene. Red dots show up-regulated genes and green dots down-regulated genes in regenerated skin (FDR < 0.05, |log_2_FC(fold change)) >1).

**Figure 3:**
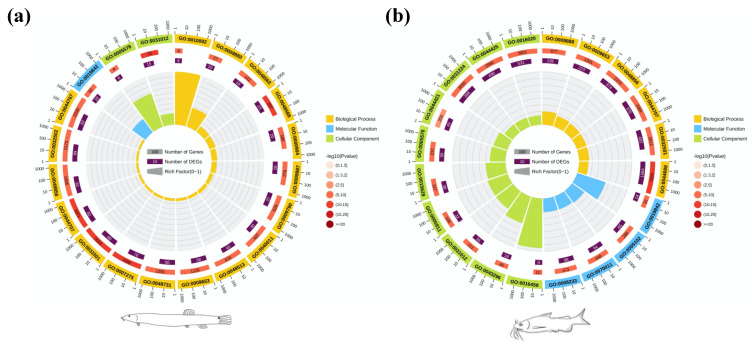
Top 20 of GO classifications enriched in the differentially expressed genes (DEGs) in comparisons of RS/OS pertain to loach (**a**) and yellow catfish (**b**). RS: regenerated skins; OS: original skins. The first circle from outside indicates gene number scale and GO items present by GO ids; Second circle indicates gene numbers under each GO item, and third circle shows enriched gene number of DEGs. The concentric circles represent rich factors of each GO item, which are equal to the ration of gene number of DEGs and background gene number under each GO item.

**Figure 4:**
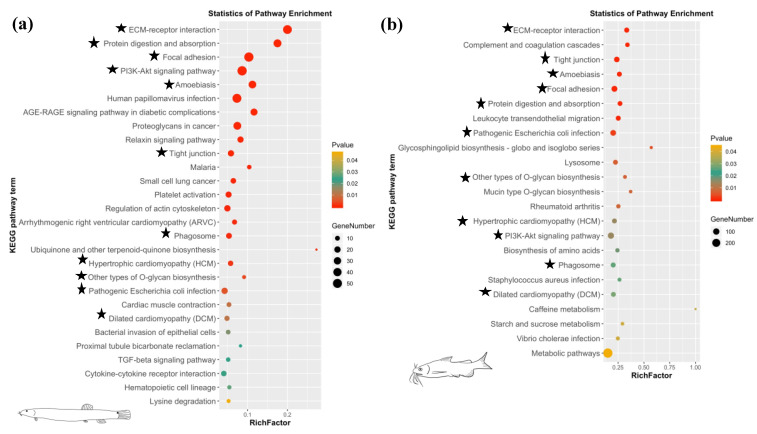
Enrichment of KEGG pathways of differentially expressed genes (DEGs) in the comparisons of RS/OS pertain to loach (**a**) and yellow catfish (**b**). RS: regenerated skins; OS: original skins. Black stars indicate common enriched KEGG pathways between loach and yellow catfish.

A total of 640 genes and 4446 genes were significant differentially expressed in the comparisons of RSs and OSs of loach and yellow catfish, respectively (Table S2). There were 460 up- and 180 down-regulated DEGs in regenerated skin in comparison of original skin of loach (Figure 2a). Meanwhile, in the regenerated skin and original skin pairwise comparison of yellow catfish, 2340 up- and 2106 down-regulated DEGs were detected in the regenerated skins of yellow catfish (Figure 2b). A total of 295 and 418 GO items were significantly enriched in the regenerated skin of loach and yellow catfish, respectively (Table S3). Among these GO items, extracellular matrix (GO:0031012), multicellular organismal process (GO:0032501), single-multicellular organism process (GO:0044707), anatomical structure development (GO:0048856), anatomical structure morphogenesis (GO:0009653), and vitamin binding (GO:0019842) were simultaneously annotated as top 20 enrichment GO items in the comparisons of RS and OS in loach and yellow catfish (Figure 3). In addition, 28 and 23 KEGG pathways were enriched among the DEGs in the comparisons of RSs and OSs pertain to loach and yellow catfish, respectively (Figure 4a,b). Among these KEGG pathways, ten KEGG pathways were simultaneously annotated as enriched pathways in both of two fishes. ECM-receptor interaction (ko04512) was annotated as highest enriched pathway in both of loach and yellow catfish. Most of the pathways were annotated to relate to cellular differentiation, proliferation, growth and development, while a few immune-related pathways were also enriched in the regenerated skin, such as bacterial invasion of epithelial cells (ko05100) in loach and staphylococcus aureus infection (ko05150) in yellow catfish.

### 3.4. Mining of Cutaneous Air-Breathing Related Genes in Loach

**Figure 5:**
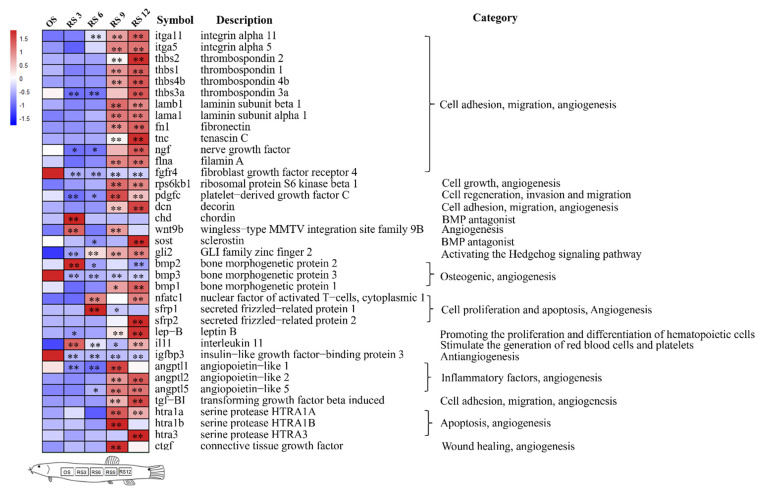
Expression profiles of cutaneous air-breathing formation related genes in regenerated skin of loach. OS: original skin; RS3: regenerated skin of the 3rd day after cutaneous incision (daci). RS6: regenerated skin of the 6th daci. RS9: regenerated skin of the 9th daci. RS12: regenerated skin of the 12th daci. * indicates significant difference of gene express level between regenerated skin and original skin (* *p* < 0.05, ** *p* < 0.01).

**Figure 6:**
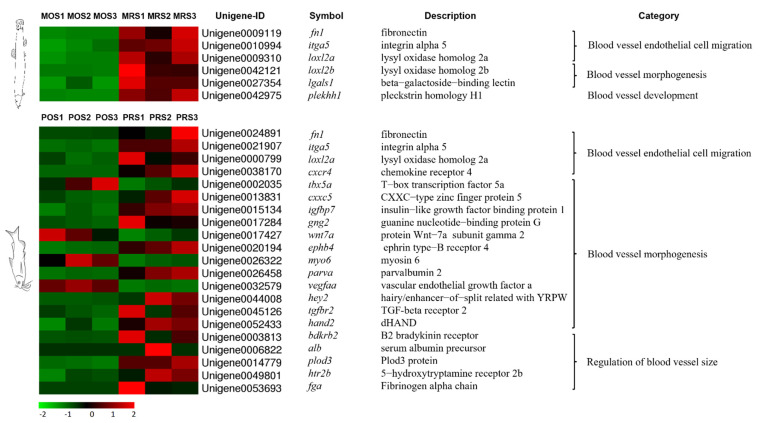
Expression performances of differentially expressed vascularization-related genes in comparisons of RS/OS pertain to loach and yellow catfish. RS: regenerated skins; OS: original skins.

Based on the annotation results of all unigenes and the expression performances in loach, 37 potential genes of cutaneous air-breathing formation were targeted (Figure 5). Among these genes, *itga11*, *itga5*, *thbs2*, *thbs1*, *thbs4b*, *fn1*, and *rps6kb1* were up-regulated, whereas *fgfr4*, *bmp3*, and *igfbp3* down-regulated upon skin regeneration; *thbs3a*, *ngf*, *pdgfc*, *sost*, *bmp1*, and *angptl1* were up-regulated and then down-regulated, while *chd* and *bmp2* presented opposite expression patterns. In addition, potential genes related to cutaneous air-breathing were excavated in the comparisons of MRS/MOS and PRS/POS. Tens of DEGs, subordinating to thirteen pathways including TGF-beta signaling pathway (ko04350), Jak-STAT signaling pathway (ko04630), Wnt signaling pathway (ko04310), vascular smooth muscle contraction (ko04270), were probably related to cutaneous air-breathing formation in loach (Figure S3, Table 2). These genes were most associated with single-organism process (GO:0044699), multicellular organismal process (GO:0032501), developmental process (GO:0032502), single-multicellular organism process (GO:0044707), and anatomical structure development (GO:0048856) (Table S4). Meanwhile, a number of DEGs related to vascular angiogenesis and development in yellow catfish were selected and illustrated in Figure 6 as well, for the purpose of further narrowing the scope of the target genes. Finally, after filtered by the data of yellow catfish, *loxl2b*, *lgals1* and *plekhh1* were considered as the key candidate genes involved cutaneous air-breathing formation in loach.

### 3.5. Validation of DEGs by qPCR

**Figure 7:**
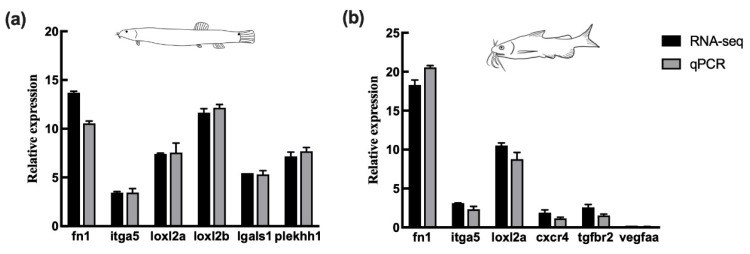
Validation of expression levels of differentially expressed genes (DEGs) in comparisons of RS/OS pertain to loach (**a**) and yellow catfish (**b**) between RNA-seq and qPCR. RS: regenerated skins; OS: original skins.

To validate the gene expression profiles identified by RNA-seq, six DEGs (*fn1*, *itga5*, *loxl2a*, *loxl2b*, *lgals1*, and *plekhh1*) from loach and six DEGs (*fn1*, *itga5*, *loxl2a*, *cxcr4*, *tgfbr2*, and *vegfaa*) from yellow catfish were chosen to do qPCR analysis. The expression patterns of these genes revealed by qPCR were rather similar to those revealed by RNA-seq (Figure 7a,b).

## 4. Discussion

In spite of slight structure differences in fish skins with accessory air-breathing function, there are some characteristics shared: thick skin layer, well differentiated gland cells, highly developed vascular network, capillary epithelialization, and reduced or even disappeared scales [20]. From the perspective of both morphology and histology, the skin of loach was fine modified for the adaption of air-breathing. The middle layer of cutaneous epidermis in air-breathing fish is composed mostly of a variety of epithelial cells with oxygen carrying capacity [19], however, this layer of loach was mainly composed of multi-layered swollen cells, which were bulky and vacuolated. Similar structures occurred in the middle layer of amphibious skin [35]. The multi-layered structure possibly played a role of a water-blocking barrier to keep the skin moist when exposed to the air for a long time [22]. In many fish species with cutaneous respiration, such as mudskipper (*Periophthalmus cantonensis*), abundant micro-vessels were also detected in the epidermis [35]. Two “trauma models” of skin resection in loach (cutaneous air-breathing fish) and yellow catfish (non-cutaneous air-breathing fish) were uniquely adopted in exploration to the mechanism of fish cutaneous accessory respiration. Based on the observation of histological structures, the capillaries appeared in the basal layer of the epidermis of original and regenerated skins to promote cutaneous air-breathing in loach. The well vascularized tissues/organs provided the structural basis for gas exchange.

After the cutaneous trauma occurs, it is repaired in three stages: inflammation, tissue formation, and tissue remodeling [36], among which angiogenesis and vascularization are important factors in wound healing [37]. The histological evidence indicated that the respiratory function of fish skin lies on the well vascularization in skin [35,38,39,40]. The original and regenerated skins of these two fish species were investigated by transcriptome analyses. A vast of genes were identified upon skin regeneration process, and abundant genes related to cellular differentiation, proliferation, growth, and development were activated to promote the completion of the regeneration of skins. In addition, the immune response was also activated since multiple immunity genes were up-regulated in the regenerated skins to resist the invasion of external bacteria or harmful substances.

In the present study, tens of genes and thirteen pathways that potentially associated with cutaneous air-breathing were identified in loach, by comparative transcriptome analyses of original and regenerated skins. Interleukins (*itga5* and *igta11*), thrombospondins (*thbs1*, *thbs2*, and *thbs4b*), and fibronectin (*fn1*) were up-regulated upon whole process of skin regeneration. Integrins, one of the important members of the cell adhesion molecule family, are a class of transmembrane glycoprotein receptors widely distributed on the cell surface. They mainly mediate the cell-to-cell interaction and extracellular matrix, and induce cellular proliferation, differentiation, migration, and survival of endothelial cells to promote angiogenesis [41,42]. The thrombospondins are mainly involved in embryonic development, wound healing, synaptogenesis, and tumor formation [43]. Previous studies have shown that thbs1 can induce apoptosis of vascular endothelial cells by activating the CD36, P53, caspase-3, and p38-MAPK pathways [44]. Thbs1 can also prevent pro-angiogenic growth factors from binding to their receptors on the surface of endothelial cells while indirectly inhibiting angiogenesis [45]. Thbs2 can bind to CD36 and inhibit the migration of vascular endothelial cells and the formation of luminal cavity [46]. The thrombospondin members of *thbs1*, *thbs2* and *thbs4b* were up-regulated at 9^th^ daci in loach, which indicated that the vascular construction of regenerated skin was initially completed, consistent with the histological observation of regenerated skin. In addition, fibronectin plays a vital role in cell adhesion [47], blood vessel development, and heart morphogenesis both in zebrafish [48] and mouse [49,50]. *fn1* was up-regulated in the regenerated skin of loach, which indicated a potential role of cutaneous air-breathing formation in loach, whereas the genes of *bmp2*, *chd*, *il11*, *wnt9b* and *nfatc1* were up-regulated at the initial stage of skin regeneration (3th and 6th daci). Bone morphogenetic protein (BMP) signaling has recently emerged as a fundamental pathway of the endothelium by regulating cardiovascular and lymphatic development and by being causative for several vascular dysfunctions [51]. It is reported that bmp2 can activate endothelial cells and significantly promote the sprouting and extension of vascular endothelial cells, thereby forming a blood vessel network [52]. Il11, a member of IL6 superfamily, has been found to have many physiological functions, such as stimulating red blood cell and platelet production [53]. Wnt signaling pathway family proteins are secreted glycoproteins that play an important role in embryonic development [54].

Considering the DEGs of regenerated skin of yellow catfish, we further screened out *loxl2b*, *lgals1*, and *plekhh1*, which were considered as the key candidate genes involved cutaneous air-breathing formation in loach. LOXL2, a prototypical lysyl oxidase, is implicated in the promotion of cancer cell invasion, metastasis and angiogenesis, as well as in the malignant transformation of solid tumors [55]. *Loxl2* increases the expression level of *vegf* in cancer, while angiogenesis contributes to primary and metastatic cancer growth, and it is necessary for tumor progression [56,57]. A study on the effects of *lox* expression on tumor-driven angiogenesis, demonstrated that the regulation of mRNA and protein expression was carried out through the platelet-derived growth factor β (PDGFRβ)-mediated activation of protein kinase B (Akt) [58]. Moreover, the inhibition of *loxl2* activity inhibited angiogenesis in part by affecting VEGF signaling in endothelial cells [59] *Lgals1*/Galectin 1 is one of the members of galectin and plays multifaceted roles in cell adhesion, proliferation, angiogenesis, and immunosuppression, targeting not only a variety of cancer cells, but also vascular endothelial cells and regulatory T cells [60,61]. *Lgals1* was reported to play a crucial role in promoting angiogenesis in anti-VEGF-A refractory tumors and was shown to interact with the N-glycans on Ig domains 3, 4, and 7 of *vegfr2*, causing increased phosphorylation of VEGFR2 and activation of its downstream signaling pathways in endothelial cells [62]. Anti-VEGF-A refractory tumors enhanced galectin-1 secretion together with its increased binding to neovascular endothelial cells due to altered glycosylation patterns on VEGFR2, leading to galectin-1-driven angiogenesis and tumor progression [63]. *Plekhh1* is a number of pleckstrin homology domain superfamily, containing two predicted coiled-coil domains and a phosphositide-binding module called PH domain, as little has been known about this protein. [64,65]. Notably, *fn1* also differentially expressed in yellow catfish, but the degree of significant difference was much less than that of loach. FN1 performs an important role in angiogenesis and development, and recent reports demonstrated the activation on Akt signaling pathway by binding with its receptor ITGA5, further activating mTOR pathway to regulate angiogenesis [66].

It is generally acknowledged that neovascularization mainly refers to the formation of blood vessels by the existing capillaries by germination, while angiogenesis mostly means the proliferation and differentiation of endothelial progenitor cells, turning into endothelial cells to form new blood vessels [67,68,69]. Actually, not only could micro-vessels provide adequate fresh blood with nutrient substances for inflammatory cells and stem cells necessary for wound repair [70], but also perform an indispensable role for aerial ventilation in loach of skin defect. Therefore, a reasonable speculation was put forward that there was a balance for blood angiogenesis and development during skin regeneration between promoting of tissue healing and maintaining the function of skin air-breathing in fishes with cutaneous respiration. Nevertheless, it is still to be examined what exact factors affecting this balance and its dynamic changes in the future exploration.

## 5. Conclusions

In the present study, we examined the skin regeneration of a cutaneous air-breathing loach *M. anguillicaudatus* and a water-breathing yellow catfish *P. fulvidraco* with respect to the morphological and histological observations and gene expression profiles. The capillaries embed in the basal layer of the epidermis of original and regenerated skins promote cutaneous air-breathing in fish, which provided the structural basis for gas exchange. Subsequently, the comparative transcriptomic analysis indicated that tens of genes and thirteen key pathways were strongly tied to cutaneous skin air-breathing in loach. This study provides substantial contribution to the molecular biology profiles of skin regeneration of fish, and, furthermore, offers new insights into the formation mechanism of cutaneous air-breathing in bimodal respiration fish.

## Figures and Tables

**Table 1:** Summary of mixed-assembling transcriptome data of loach *M. anguillicaudatus* and yellow catfish *P. fulvidraco*.

Items	Loach	Yellow Catfish
Total length	36.07 Gb	44.83 Gb
Total number of clean reads	266,126,010	291,478,754
Total number of unigenes	56,056	53,731
Mean length of unigenes (nt)	991	1080
Total number of N50	1922	2073

**Table 2:** Key pathways associated with cutaneous air-breathing formation in loach.

ID	Description	*p* Value	DEGs
ko04512	ECM-receptor interaction	6.36 × 10^−32^	*fn1*, *itga11*, *itga5*, *lama1*, *lamb1*, *lamc3*, *thbs1*, *thbs2*, *thbs3a*, *thbs4b*, *tnc*
ko04510	Focal adhesion	1.58 × 10^−21^	*flna*, *fn1*, *itga11*, *itga5*, *lama1*, *lamb1*, *lamc3*, *mylpf*, *pdgfc*, *thbs1*, *thbs2*, *thbs3a*, *thbs4b*, *tln2*, *tnc*
ko04810	Regulation of actin cytoskeleton	9.06 × 10^−4^	*itga5*, *itga11*, *fn1*, *pdgfc*, *mylpf*, *bdkrb2*, *fgfr4*, *tmsb*
ko04350	TGF-beta signaling pathway	2.42 × 10^−2^	*bmp2*, *rps6kb1*, *thbs1*, *dcn*, *nbl1*
ko04630	Jak-STAT signaling pathway	3.28 × 10^−1^	*lep-B*, *il11*
ko04310	Wnt signaling pathway	6.18 × 10^−1^	*nfatc1*, *sfrp1*, *serpinf1*, *sfrp2*, *fzd2*
ko04115	p53 signaling pathway	7.47 × 10^−1^	*thbs1*, *igfbp3*
ko04340	Hedgehog signaling pathway	8.68 × 10^−1^	*bmp2*, *ihhb*
ko04010	MAPK signaling pathway	8.82 × 10^−1^	*nfatc1*, *fgfr4*, *flna*, *cacna1b*, *ngf*, *cacnb4*
ko04270	Vascular smooth muscle contraction	9.31 × 10^−1^	*myl6*, *prkg1*
ko04150	mTOR signaling pathway	9.60 × 10^−1^	*rps6kb1*
ko04012	ErbB signaling pathway	9.60 × 10^−1^	*rps6kb1*
ko04910	Insulin signaling pathway	9.70 × 10^−1^	*rps6kb1*, *fbp2*

## Data Availability

All sequencing data are deposited in the DNA Data Bank of Japan (DDBJ) database under accession DRA012772.

## Supplementary Materials

The following are available online at https://www.mdpi.com/article/10.3390/biology10121294/s1, Figure S1: Confirmation experiment of cutaneous air-breathing in loach. Figure S2: Detections of homologous genes in five public databases (Nr, Swiss-Prot, KEGG, KOG, and GO) for loach and yellow catfish. Figure S3: Network of KEGG pathways and target genes associated with cutaneous air-breathing formation in loach. Table S1: Primer sequences used in the present study for quantitative PCR (qPCR). Table S2: Differentially expressed genes in comparisons of RS/OS pertain of loach and yellow catfish. Table S3: Enriched GO items of DEGs in comparisons of RS/OS pertain of loach and yellow catfish. Table S4: Key GO items associated with cutaneous air-breathing formation in loach.
