# Supplementary material for: Comparative Transcriptomic Analysis of Regenerated Skins Provides Insights into Cutaneous Air-Breathing Formation in Fish
Source: Biology (Basel). 2021 Dec 8;10(12):1294. doi: 10.3390/biology10121294 (PMC8698756; doi:10.3390/biology10121294)
Supplement: Supplementary file 1 [file biology-10-01294-s001.zip › Supplemental Materials/supplemental materials.pdf]

Comparative transcriptomic analysis of regenerated skins provides insights into cutaneous air–breathing formation in fish

Songqian Huang[1,2,†], Bing Sun[1,†], Longfei Huang[1,3], Lijuan Yang[1], Chuanshu Liu[1], Jinli Zhu[4], Jian Gao[1,3*], Xiaojuan Cao[1,3*]

[1]Key Lab of Freshwater Animal Breeding, Ministry of Agriculture, College of Fisheries, Huazhong Agricultural University, Wuhan 430070, China.

[2]Department of Aquatic Bioscience, Graduate School of Agricultural and Life Sciences, The University of Tokyo, Bunkyo–ku, Tokyo 113–8657, Japan.

[3]Engineering Research Center of Green development for Conventional Aquatic Biological Industry in the Yangtze River Economic Belt, Ministry of Education/Hubei Provincial Engineering Laboratory for Pond Aquaculture, College of Fisheries, Huazhong Agricultural University, Wuhan 430070, China.

[4]National Demonstration Center for Experimental Aquaculture Education, Huazhong Agricultural University, Wuhan 430070, China.

† means co–first author.

* Co–corresponding author: gaojian@mail.hzau.edu.cn (J.G.); caoxiaojuan@mail.hzau.edu.cn (X.C.). Tel.: +86(027)87282113; Postal address: No.1 Shizishan Stress, Hongshan District, Wuhan 430070, Hubei Province, China.

Supplemental materials:

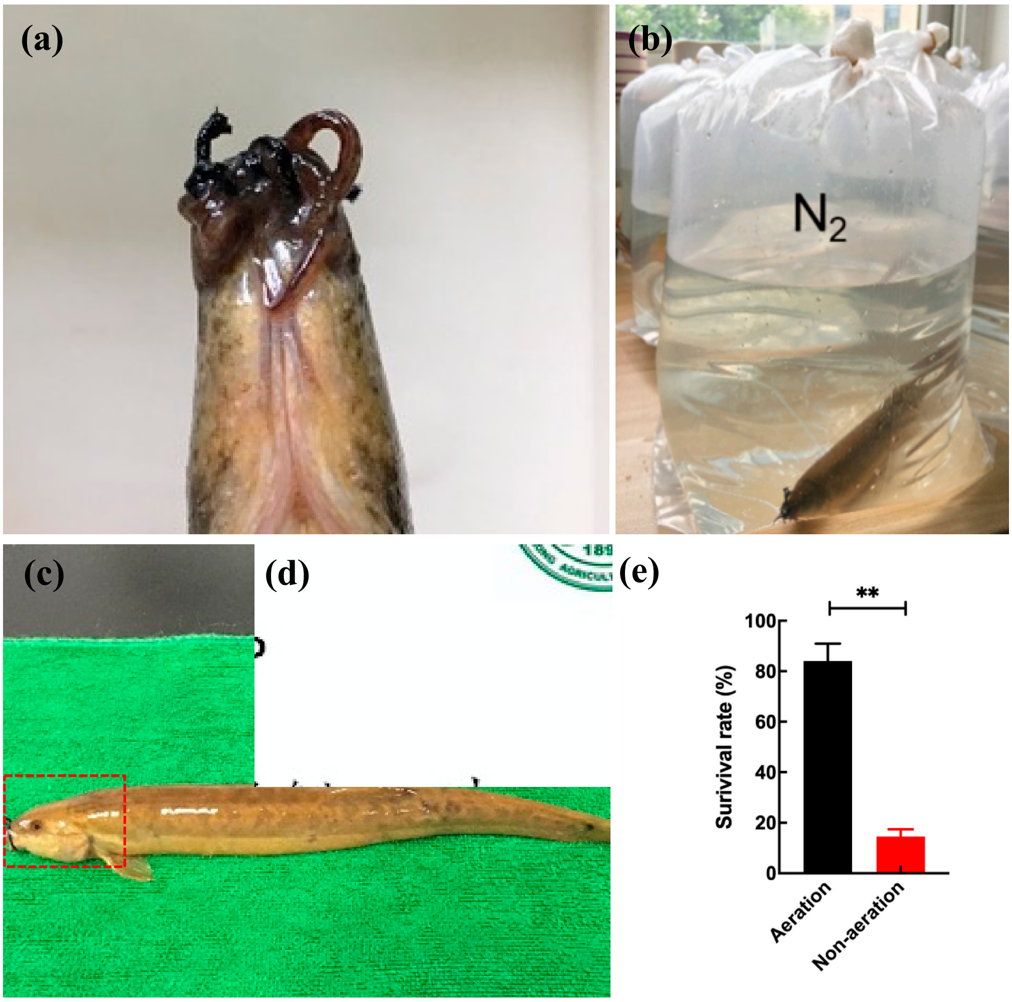

Figure S1. Confirmation experiment of cutaneous air–breathing in loach. **(**a) Loaches mouth was sutured with surgical thread. (b) Loaches with mouth stitched were placed in water in a closed plastic bag (2/3 volume was water and 1/3 was nitrogen). (c) After asphyxia by hypoxia, half of the loaches with mouth stitched were exposed to the air (the air exposure group). (d) Vision in the red dashed frame of c. (e) Survival rates of loaches from the air exposure group (Aeration) and the control group (Non–aeration) at 15 minutes after air exposure. ** $P < 0.01$.

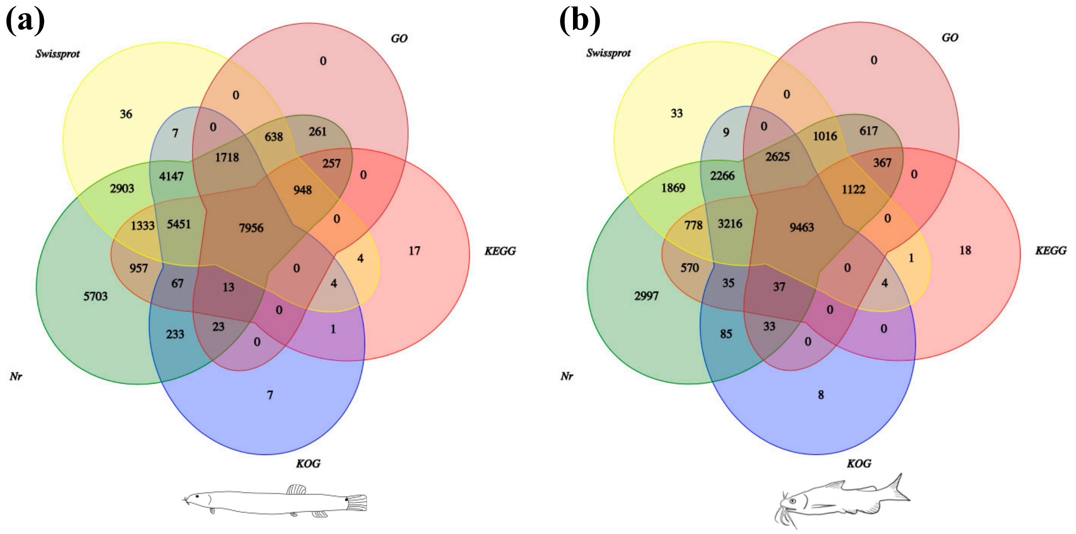

Figure S2. Detections of homologous genes in five public databases (Nr, Swiss–Prot, KEGG, KOG and GO) for loach (a) and yellow catfish (b).

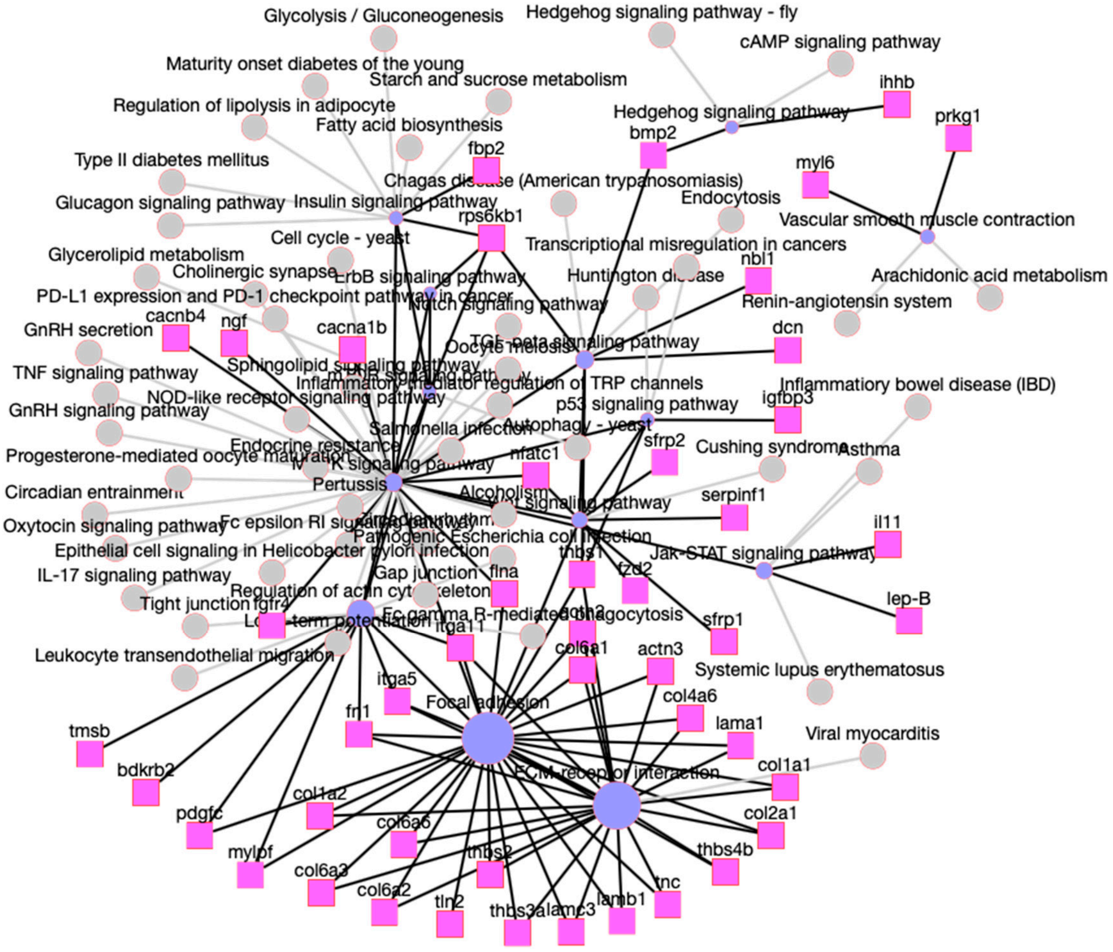

Figure S3. Network of KEGG pathways and target genes associated with cutaneous air–breathing formation in loach. Blue circles showed enriched KEGG pathways of DEGs, grey circles showed relevant pathways of these enriched pathways, and pink panes indicated cutaneous air–breathing formation related genes.

Table S1 Primer sequences used in the present study for quantitative PCR (qPCR)

Species	Gene	Forward primer (5'–3')	Reverse primer (5'–3')
Loach	*angptl1*	CGAGTATGGAGGCGGCTTCTAC	GCATCTTGTGGCGGACTTCAGT
	angptl2	CAGCGATGAAGGTCCTAAGGTTGAA	ACGATTCTCCAGCACAGCCTCT
	angptl5	TCGCAAGATGATTCCACAGCCTATG	GCCATCATTGTCAACATCGGAGGTA
	bmp1	GAGGAGGCTGATTGTGGCTATGATT	CCTTCTTGTTGATGGTGTCGTCTGA
	bmp2	ATTCCGCAGTACATGCTGGACTTGT	TCCGCTGATGTCACCGACTCCT
	bmp3	CCTCGTAAGAAGCCTCGCAAGAAC	AGATAATCCACTCGCTCCAGCCAAT
	chd	GCCTGCCGAAACATACCAACCT	TCCTCTCAAACTCTCGTCTTCCTCT
	ctgf	TGGATTCAGACTACGCAACGAGAT	CTAAGAAGGTCAGGCACAGGAGAG
	dcn	AATGAGATCAGCGTGGTGGAGAATG	TGTTCCTATGGCGGCAATCTTGTT
	fgfr4	ACAATGGAAGATGCTGGCGAATACA	GCGGCAGAGAATCACGATAACGAT
	flna	ATTGTTCCTGTTGCATCGCTGTCA	AATCGCTCCTCTCGCTCCATTCA
	fn1	ACCAATCACGAGGTCACAGATGTTC	CCGATGTCTCAGTCTCAGGAAGGA
	gli2	CAGGACTCGCATGACATCAACAAC	TGAACTTGGACACTGGCAGACTG
	htra1a	TCGCTCGCTTGACAGATTATTCCA	GCAACGACGGTAAGACGCAGAT
	htra1b	GTCTGGAGTGCGTGGTGTCTGA	GGATGAAGATGATAGGCGGCTGTTG
	htra3	CGATGTGAGCAAGTGTCCGAGTC	TGGCAGATGTTACCGTATGTGTTCC
	igfbp3	CCAAGAGGCTTCCGCATTCCAA	TTGTCCACGCACCAGCAGTAAC
	il11	ACTGTCTTCCATCTTCGTTGCTCTC	GTGACTCCTCTTCGCTGCTCCT
	itga11	TGAGAAGGTGTCTGGTCTGATTGGT	GTCATCACTGCTGGCTCTTCTTCTC
	itga5	CTCCACCGCTGCTGTTATCATCAA	TCTGCCGTCTGGTAGTTGAGGAT
	lama1	TGACGCTCGCTGGCACTCTAT	GGATTCTCTGTGGTGACGGTAACG
	lamb1	GCCAACACAATGCCTGATGATGAC	TGAGAGCCGAGTAGAGCGAGAG
	lep–B	TTGCCTGCTCTCATCTACACCTG	CGGATTCTGGAGTTGATGGTCTCT
	lgals1	AGACTTGACCAGAATGAGAACA	AGCGAGTAGGTGGCGGATGTT
	loxl2a	TGTTCCGCTTCTCCTCGCAGAT	GCAGCCGACTGTGATTCCTTGT
	loxl2b	GCTTCTGTCTGGAGGATTCTG	TGTCTTCTGTCTCGGCACTAA
	nfatc1	AACAGAGCCGCACGACGACTA	GAGGACGAACAGGACAAGGAGGT
	ngf	CGATCCGAAGAACAGAGCCAAGAG	TACCGTGACCTCGTTGCCAGAA
	pdgfc	CCAACACATATCCTCGCAATACAGT	GGCTCCTCCACTTCCACATAATCA
	plekhh1	AGCGAGTAGGTGGCGGATGTT	TAACGACGGAGCGATGCGAATG

	rps6kb1	GAACAGAGCAGATTCGTCCTGAGTG	GTGAGCCGTGTCCTTCGCATT
	sfrp1	AACTTACTGGAGCACGAGACTATGG	GCAAAGAGGGCACACAGAAACAC
	sfrp2	CTGTATGCTCTGTCTCTGCTGAAGT	GACCGAAGGCGTATAATCCGTGAA
	sost	GGTGGAGAAGCAATTCATCCGACTA	CGATTGGTTGTGATGGCGAGTGTA
	tgf–BI	GCACAGTTCTGGAGGTCGGTTG	GGTCACAGACTCAGAGCCAATAAGG
	thbs1	CTGCTGTGGACGATGAATGAAGGA	GCTCTGGCTGTGTCAATATCAATGC
	thbs2	CGAACCAAGCCGACCACGAT	GCATCACCACGACCATCACCAT
	thbs3a	GGTCGGTCTGTTCGTGTTGATGTT	TTAGGTGGCAGGCGGAAGTTAGA
	thbs4b	GGAGACGAATGTGACGAGGATGATG	ACGACTGTCTGATAGGCTCTGAAGT
	tnc	CTACGGACTGCTCACAGACTCTACT	GACGCCTCAGAATCACCATCCATC
	wnt9b	CCTGAAGCAATGCGAGCAGATGA	GTTACGGAACTGATAGCGGCACTC
	wnt9b	CCTGAAGCAATGCGAGCAGATGA	GTTACGGAACTGATAGCGGCACTC
Yellow catfish	*fn1*	CCTCGTCGTGTTCGGATCTCCA	TGGTGGCAGCAATGGTGAATGG
	itga5	GCCAGGTCCTCCAAGCACTACT	CAGCCAGCCGAACATCACAGAG
	loxl2a	CCTCCACTCGCACCTCCAGAAT	CGACCTTAACGCCGCACAAGT
	cxcr4	ATGGACTGGTGGTGCTGGTGAT	AGGCTGATGAAGGCGAGGATGA
	vegfa	CCAACCGTCTCAGGCAGCAAT	CGAGTGTGAGCGAGCAAGTCTT
	tgfbr2	GCCTCTCGCTGTGATGTTATAG	GTTCTTCCTGTGCCATAGTGTT
	β–actin	GCCGTGACCTGACTGACTACCT	AGAGGAGGAAGAGGCAGCAGTG

angptl1: angiopoietin–like 1; *angptl2*: angiopoietin–like 2; *angptl5*: angiopoietin–like 5; *bmp1*: bone morphogenetic protein 1; *bmp2*: bone morphogenetic protein 2; *bmp3*: bone morphogenetic protein 3; *chd*: chordin; *ctgf*: connective tissue growth factor; *cxcr4*: chemokine receptor 4; *dcn*: decorin; *fgfr4*: fibroblast growth factor receptor 4; *flna*: filamin A; *fn1*: fibronectin; *gli2*: GLI family zinc finger 2; *htra1a*: serine protease HTRA1A; *htra1b*: serine protease HTRA1B; *htra3*: serine protease HTRA3; *igfbp3*: insulin–like growth factor–binding protein 3; *il11*: interleukin 11; *itga11*: integrin alpha–11; *loxl2*: lysyl oxidase–like 2; *itga5*: integrin alpha 5; *lama1*: laminin subunit alpha–1; *lamb1*: laminin subunit beta 1; *lep–B*: leptin B; *lgals1*: lectin galactoside–binding–like 1; *nfatc1*: nuclear factor of activated T–cells, cytoplasmic 1; *ngf*: nerve growth factor; *plekhh1*: pleckstrin homology H1; *pdgfc*: platelet–derived growth factor C; *rps6kb1*: ribosomal protein S6 kinase beta–1; *sfrp1*: secreted frizzled–related protein 1; *sfrp2*: secreted frizzled–related protein 2; *sost*: sclerostin; *tgf–BI*: transforming growth factor beta induced; *thbs1*: thrombospondin–1; *thbs2*: thrombospondin–2; *thbs3a*: thrombospondin 3a; *thbs4b*: thrombospondin 4b; *tgfbr2*: transforming growth factor–beta receptor 2; *tnc*: tenascin C; *vegfa*: vascular endothelial growth factor a; *wnt9b*: wingless–type MMTV integration site family 9B.

Table S2. Differentially expressed genes in comparisons of RS/OS pertain of loach and yellow catfish.

Table S2.xlsx

Tables S3. Enriched GO items of DEGs in comparisons of RS/OS pertain of loach and yellow catfish.

Table S3.xlsx

Table S4. Key GO items associated with cutaneous air–breathing formation in loach

ID	Class	Description	Pvalue	DEGs
GO:0044699	BP	single–organism process	1.69×10^{-2}	*lamb1, lama1, itga5, itga11, fn1, dcn, lep-B, tnc, fzd2, fgfr4, ihhb, flna, tmsb, bdkrb2, sfrp1, sfrp2, pdgfc, igfbp3*
GO:0032501	BP	multicellular organismal process	1.85×10^{-12}	*lamb1, lama1, itga5, fn1, dcn, lep-B, tnc, fzd2, ihhb, tmsb, bdkrb2, sfrp1, sfrp2, igfbp3*
GO:0032502	BP	developmental process	1.31×10^{-10}	*lamb1, lama1, itga5, fn1, dcn, lep-B, tnc, fzd2, ihhb, tmsb, nbl1, pdgfc, thbs4b, igfbp3*
GO:0044707	BP	single-multicellular organism process	3.76×10^{-11}	*lamb1, lama1, itga5, fn1, dcn, lep-B, tnc, fzd2, ihhb, tmsb, sfrp1, sfrp2, igfbp3*
GO:0048856	BP	anatomical structure development	1.01×10^{-10}	*lamb1, lama1, itga5, fn1, dcn, lep-B, tnc, fzd2, ihhb, tmsb, nbl1, thbs4b, igfbp3*
GO:0044767	BP	single-organism developmental process	3.22×10^{-9}	*lamb1, lama1, itga5, fn1, dcn, lep-B, tnc, fzd2, ihhb, tmsb, pdgfc, igfbp3*
GO:0007275	BP	multicellular organism development	5.02×10^{-12}	*lamb1, lama1, itga5, fn1, dcn, lep-B, tnc, fzd2, ihhb, tmsb, igfbp3*
GO:0009653	BP	anatomical structure morphogenesis	2.56×10^{-10}	*lamb1, lama1, itga5, fn1, dcn, tnc, fzd2, ihhb, tmsb, nbl1, igfbp3*
GO:0048731	BP	system development	2.21×10^{-9}	*lamb1, lama1, itga5, fn1, dcn, lep-B, tnc, fzd2, ihhb, tmsb, igfbp3*
GO:0005515	MF	protein binding	2.44×10^{-5}	*lama1, itga5, fn1, dcn, tln2, tmsb, pdgfc, thbs4b, igfbp3*
GO:0016043	BP	cellular component organization	1.01×10^{-2}	*lamb1, lama1, itga5, fn1, tnc, fzd2, tln2, tmsb,*
GO:0071840	BP	cellular component organization or biogenesis	1.62×10^{-2}	*lamb1, lama1, itga5, fn1, tnc, fzd2, tln2, tmsb,*
GO:0048513	BP	animal organ development	9.70×10^{-9}	*lamb1, lama1, fn1, dcn, lep-B, fzd2, ihhb, tmsb,*

BP: Biological Process; MF: Molecular Function.
